# Smartphone-Based Quantitative Analysis of Protein Array Signals for Biomarker Detection in Lupus

**DOI:** 10.3390/chemosensors10080330

**Published:** 2022-08-13

**Authors:** Guang Yang, Yaxi Li, Chenling Tang, Feng Lin, Tianfu Wu, Jiming Bao

**Affiliations:** 1 Materials Science & Engineering, University of Houston, Houston, TX 77204, USA; 2 Department of Biomedical Engineering, University of Houston, Houston, TX 77204, USA; 3 Department of Electrical and Computer Engineering, Texas Center for Superconductivity (TCSUH), University of Houston, Houston, TX 77204, USA

**Keywords:** fluorescent microarray, smartphone application, clinical diagnostics, biomarker, image processing

## Abstract

Fluorescence-based microarray offers great potential in clinical diagnostics due to its high-throughput capability, multiplex capabilities, and requirement for a minimal volume of precious clinical samples. However, the technique relies on expensive and complex imaging systems for the analysis of signals. In the present study, we developed a smartphone-based application to analyze signals from protein microarrays to quantify disease biomarkers. The application adopted Android Studio open platform for its wide access to smartphones, and Python was used to design a graphical user interface with fast data processing. The application provides multiple user functions such as “Read”, “Analyze”, “Calculate” and “Report”. For rapid and accurate results, we used ImageJ, Otsu thresholding, and local thresholding to quantify the fluorescent intensity of spots on the microarray. To verify the efficacy of the application, three antigens each with over 110 fluorescent spots were tested. Particularly, a positive correlation of over 0.97 was achieved when using this analytical tool compared to a standard test for detecting a potential biomarker in lupus nephritis. Collectively, this smartphone application tool shows promise for cheap, efficient, and portable on-site detection in point-of-care diagnostics.

## 1. Introduction

Chronic kidney diseases (CKD), such as lupus nephritis (LN), have become a significant societal and financial burden globally [1,2]. Effective clinical management of patients with chronic conditions largely relies on early diagnosis and timely monitoring of these diseases. Given the increasing population of CKD patients and corresponding reduction of medical resources per patient, the current clinics-based healthcare system is facing great challenges in effectively tracking the disease status of these patients [3]. Point-of-care systems are emerging healthcare technologies and may be promising in opening up a new avenue in tackling these challenges [4]. Blood glucose meters [5] and lateral flow assay (LFA) based pregnancy tests [6] represent so far the most successful point-of-care systems to detect single disease markers. However, for complicated chronic diseases such as lupus, lupus nephritis, or other CKDs, multiple disease markers are needed for an accurate diagnosis. Microarray technology provides a new opportunity to develop low-cost, high-throughput, multiplexing, and minimal-volume detection assays for clinical and point-of-care diagnostics [7-12], a laboratory-based, computerized microarray scanner is large, expensive, and not suitable for point-of-care testing.

Since smartphone is a cost-effective and portable device with high-quality digital cameras, excellent computing ability, touchscreen interface, and wireless data transfer capability [13], it can be used as a platform for capturing images/data, analyzing and reporting results [14]. In recent years, simple mobile devices have been widely adopted for applications in food pollution monitoring, such as allergens or hormones [7,15], environmental monitoring such as metal ions in water [16,17], health status monitoring [18], and cell counting [19]. Smartphone has also been increasingly used for point-of-care diagnostics, especially early detection of biomarkers through fluorescent protein microarrays [7,8,10,13,20-23]. However, most smartphone Apps only utilize their cameras to take images, and then send the data to computer or remote server to analyze images and produce diagnosis reports [8,10,20-24]. Although some Apps are equipped with fully functional software to perform image processing and data analysis, and have demonstrated stand-alone diagnostic ability, these Apps are not freely downloadable, and there is still no open-source code available to the community [7,13,25]. Furthermore, these Apps are not flexible and optimized to handle different types and sizes of images [7,13,25]. For example, these Apps can only handle undistorted fixed array of protein images, and it is a challenge for them to read non-circular protein dots and remove uneven background fluorescence.

In this work, we demonstrated a new smartphone App using Python on the Android Studio platform to efficiently process large-size image data of protein microarrays. The App allows users to define column and row numbers of arrays so that different size of image arrays can be tested. The App uses Otsu binarization and local thresholding to localize spots, remove non-uniform fluorescence background and re-adjust fluorescence signal. Since fluorescence data point is selected by local threshold, protein dots with arbitrary shapes can be read. Since a variety of imaging accessories have been demonstrated [7,8,10,13,20-25], we will use images directly from a commercial scanner (GenePix Microarray Scanner). The images are transferred to smartphone via a cable to guarantee security, and direct processing can be realized when the images are captured by smartphone in the future. Various antigens and antibody microarrays in lupus are tested by the App with a positive correlation of over 0.95 to the data obtained from the commercial scanner. This App fills a critical need for a flexible, reliable, and open-source code for smartphone applications, will pave the way for wider application of smartphone for point-of-care diagnosis.

## 2. Materials and Methods

### 2.1. Materials

#### 2.1.1. Antigens, Antibodies, and Reagents

In our previous studies, both fluorescence-based antigen and antibody arrays were successfully developed for biomarker detection in autoimmune diseases [26-29]. Five antigens were used for the antigen array in this study. Phosphate-buffered saline (PBS) was used as a negative control, and immunoglobulin G (IgG) was used as a positive control. Centromere protein B (CENP-B, catalog no. 12500) and Ribosomal phosphoprotein P2 (P2, catalog no. 14300) were purchased from Diarect Ag (Freiburg im Breisgau, Germany), and Cytokeratin 6 protein (catalog no. ABIN2712834) was purchased from Antibodies.com (Cambridge, UK), Gastric Parietal Cell (GPC) (catalog no. ATP01-02) was from Arodia (Wellington, New Zealand), Proteoglycan (catalog no. ATP01-02) was from Sigma-Aldrich (St. Louis, MO, USA), and the positive control IgG (catalog no. P525-3) was purchased from BBI Solutions (Portland, ME, USA).

For the detection of these antigens, Cy3-conjugated goat anti-human IgG (catalog no. 109-165-088) was purchased from the Jackson ImmunoResearch (West Grove, PA, USA). The human CD14 DuoSet ELISA kit (catalog no. DY383) was purchased from R&D Systems (Minneapolis, MN, USA) for fabricating the CD14 antibody array. Serum samples were diluted with Super G blocking buffer (catalog no. 105101) from Glace Bio-Labs (Bend, OR, USA).

#### 2.1.2. CD14 Detection on an Antibody Array

The CD14 capture antibody was diluted to 240 μg/mL with PBS and was spotted in five replicates within each subarray on a slide with epoxy modification of the surface (STRATEC, Birkenfeld, Germany). After blocking with 5% bovine serum albumin (BSA) in PBS, the serial diluted human CD14 standard and diluted serum samples from lupus nephritis and healthy subjects were both incubated in a separate subarray for 2 h at room temperature. After washing each subarray with tris-buffered saline with 0.1% Tween 20 detergent (TBST), 1 μg/mL of biotinylated Anti-Human CD14 detection antibody was added to each subarray for 1 h at room temperature. Then the slide was washed with TBST, and 1 μg/mL streptavidin-Cy3 was added into each subarray for 45 min at room temperature before scanning with a GenePix microarray scanner. All human subject-related procedures were performed following the institutionally approved IRB protocol (the University of Houston, IRB# STUDY00001299), and all consents were obtained before sample collection. Given this is a proof-of-concept pilot study, serum samples from two healthy subjects and two lupus nephritis patients (with high disease activity, SLEDAI = 12) were used for the protein microarray analysis.

#### 2.1.3. Antigen Array

The antigens were serially diluted five times using 0.01M PBS as follows: CENP-B from 50 μg/mL to 3.125 μg/mL, Cytokeratin-6 from 12.5 μg/mL to 0.78 μg/mL, GPC from 12.5 μg/mL to 0.78 μg/mL, P2 from 100 μg/mL to 6.25 μg/mL, Proteoglycan from 50 μg/mL to 3.125 μg/mL, and positive control IgG from 50 μg/mL to 3.125 μg/mL. Meanwhile, 0.01 M PBS was used as a negative control. Therefore, a total of 31 antigen samples at different concentrations were prepared for fabrication of the antigen array. These antigens were spotted on a plastic microarray slide with an epoxy modification of the surface (CL01 CCO E-Coated slide from Sony, Bothell, WA, USA). The 31 antigens were loaded into a 384-well plate (Thermo Scientific, Waltham, MA, USA) and repeatedly printed for 16 fields arranged by 2 × 8 on the slide, and the assay method used for this array was the same as previously described [30].

**Figure 1.**
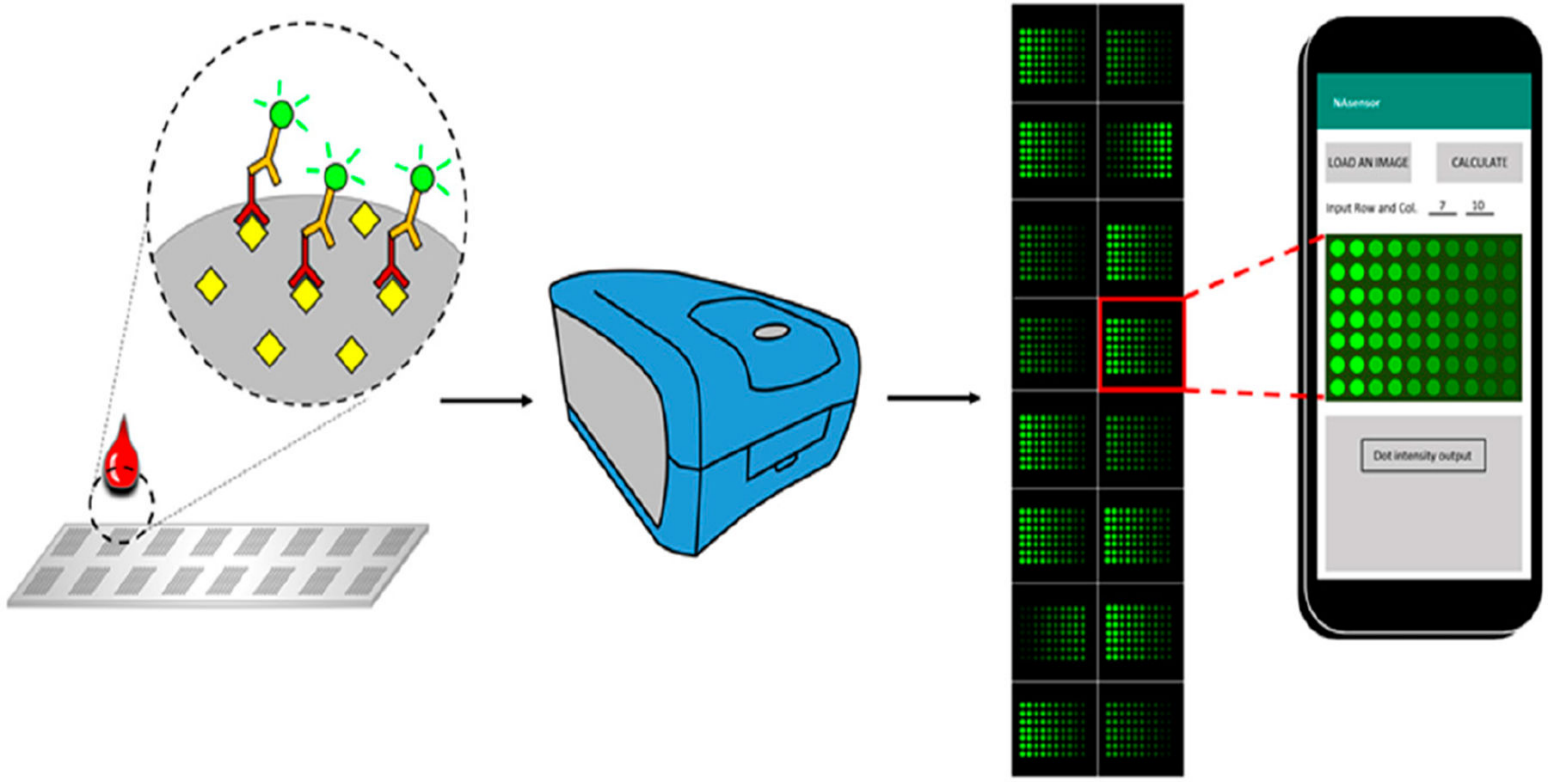
Schematic diagram of the smartphone-based imaging analysis for the fluorescent-labeled autoantibody array. Antigens were printed on the surface of an array slide, and the slide can be divided into multiple fields according to the printing design. A whole field contains hundreds of different antigen spots that are capable of detecting abundant types of corresponding autoantibodies in the serum, with each spot representing one type of antigen. Cy-3 labeled anti-human IgG was used to recognize the antigen-antibody complex, and an image was obtained through the fluorescent scanner from Molecular Devices. Subsequently, the image was then analyzed by the application in the smartphone, and data for each spot can be exported for further exploration.

The schematic diagram of the whole process for the antigen array was shown in Figure 1. After the antigens were printed, the slides were placed in a laminar flow hood to dry for 2 hrs. All 16 chambers on the assembled glass slide were blocked with 100 μL of blocking buffer at room temperature for 1 h. Then the blocking buffer was decanted, and the diluted serum samples were added into appropriate wells. The slide assembly was placed at 4 °C for overnight incubation. Following several wash steps, Cy-3 labeled anti-human IgG was added into each chamber to detect the antigen-autoantibody complexes, and a strong fluorescent signal represents a high expression level of autoantibody that corresponds to the spotted antigen.

### 2.2. Instruments and Software

The fluorescent microarray images were obtained by a commercial laboratory scanner (GenePix Microarray Scanner 4400A, Molecular Devices LLC, San Jose, CA, USA). The corresponding analysis software of the scanner is GenePix Pro 7 (Molecular Devices LLC, San Jose, CA, USA). ImageJ 1.51n was used to process the images with various thresholding methods. GraphPad Prism 8 was applied to analyze the correlation of fluorescent intensity between the laboratory scanner and the smartphone application. The smartphone application was developed based on Android Studio 3.6.1 and installed on a Huawei Honor V 20 with Android version 10.

### 2.3. Image Analysis

**Figure 2.**
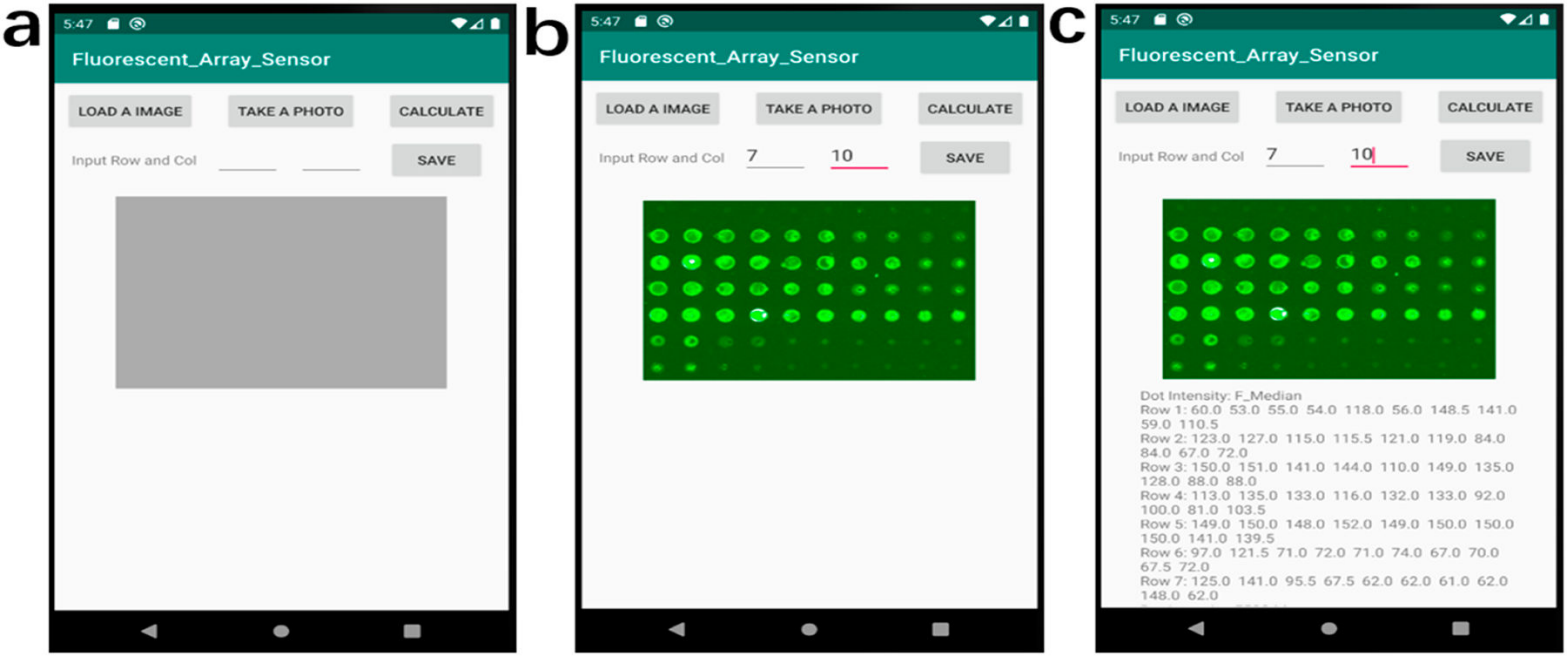
The image processing flow of the smartphone Fluorescent_Array_Sensor application. (**a**) The home page of the application. (**b**) The snapshot of the work page after loading an image and inputting the values of rows and columns. (**c**) The snapshot of the work page after calculating the data values.

To process the images of the experimental fluorescent antigen array on the smartphone, we developed a custom Android application, called Fluorescent_Array_Sensor (Permalink: https://github.com/ygsaber/Fluorescent_Array_Sensor, accessed on 9 August 2022), by using the latest free software Android Studio 3.6.1 provided by Google and JetBrains. The original fluorescent images were acquired by a laboratory scanner. The function of this image processing application is to calculate the values of the fluorescent intensity for every spot of the fluorescent antigen array in a standardized and systematic way. The determination of luminance values was based on the pixel analysis of the images. Several image-processing steps were conducted to derive the final values of the fluorescent intensity of every spot. Firstly, the uploaded 8-bit colorful image was converted to the 8-bit grey image with a weight ratio of 0.3: 0.59: 0.11 for Red: Green: Blue, respectively [31]. Secondly, the user inputs the indices according to the rows and columns of the fluorescent array in the analyzed image. Next, the grey image was divided into multiple small areas with only one fluorescent spot in each part. Then, the image of each area was converted to a binary image with only foreground and background by the Otsu thresholding method [32], Finally, the fluorescent intensity value of every fluorescent spot was calculated based on the pixel intensity. The intensity of every fluorescent spot is shown in the text area below the analyzed image. The image processing flow and user interface of the smartphone application are shown in Figure 2.

### 2.4. Statistical Analysis

The original sample images were acquired with a laboratory scanner, and then the fluorescent intensity values of every spot in the microarray images were analyzed and quantified by affiliated software GenePix Pro 7 and our smartphone application, respectively. The nonparametric Spearman’s rank correlation coefficient was calculated to reveal a possible statistical correlation between two data sets as the Equation (1) below [33]:

(1) $$r=1-\frac{6\sum d^{2}}{N(N^{2}-1)}$$

where *d* is the ranked difference between the data obtained by the smartphone and the laboratory instrument, and *N* is the number of obtained data. Among typical correlation coefficients, the Spearman’s rank correlation coefficient is selected because it evaluates a general monotonic relationship between two variables and the relationship between the two data sets is not known beforehand. The correlation analysis was performed by GraphPad Prism 8. The Spearman’s correlation (*r*) has a range from −1 to 1, and *r* = 0 means that the two compared results have no relationship, but *r* = −1 or 1 means a completely negative or positive relationship, respectively. Furthermore, the significance of the correlation coefficient was calculated to show whether the correlation relationship was reliable or not in the confidence intervals.

## 3. Results and Discussion

The image processing workflow of the smartphone application is shown in Figure 2. The starting page of the application has 4 buttons including two input texts, a grey area reserved for the image display, and a blank area reserved for the intensity results as shown in Figure 2a. After loading an image or taking a photo by clicking the corresponding buttons, the image is displayed in the grey area of Figure 2b. Then the user needs to input the values of rows and columns of the uploaded image before clicking the *‘CALCULATE’* button to analyze the image. The fluorescent intensity value of each spot was processed immediately and displayed below the image, as shown in Figure 2c. After clicking the *‘SAVE’* button, the resulting data can be saved to the smartphone.

**Figure 3.**
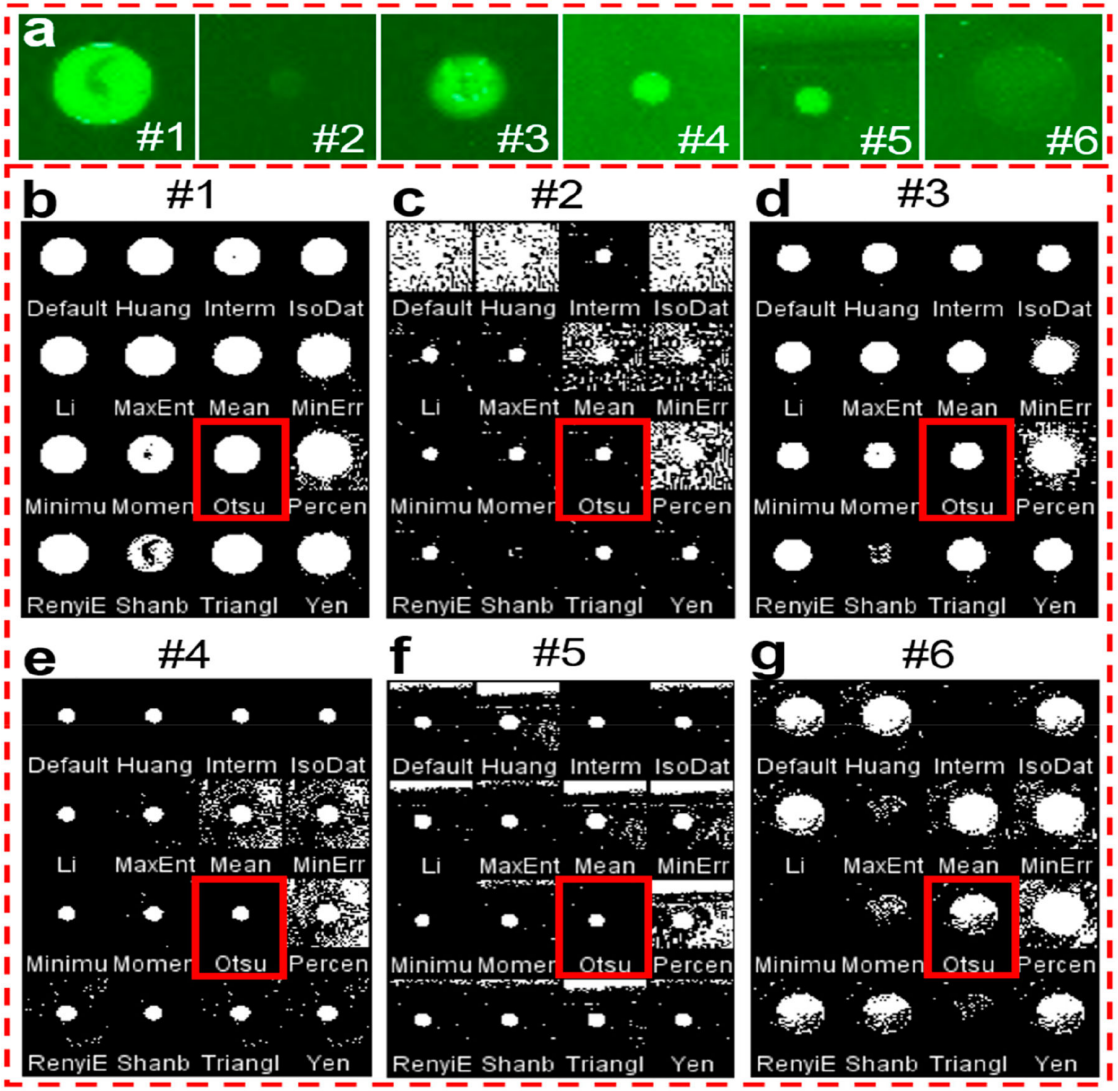
Comparison of the binary images obtained with various thresholding methods. (**a**) 6 typical fluorescent spots with different sizes, intensities, and background. (**b**–**g**) The corresponding binary images of 6 fluorescent spots after 16 thresholding methods. The red rectangles indicate the results of the Otsu thresholding method.

To obtain the intensity value of an object from its background, it is transformed into a binary image first. When considering the difference in antigen concentration, antigen types, and printing quality of each spot on the microarray, it is critical to select suitable binarization methods to acquire accurate results with the smartphone application. Here, we selected 16 typical thresholding methods to compare the processing results with 6 fluorescent spots (#1 to #6) with different spot sizes, intensities, and backgrounds (Figure 3a). Figure 3b-g shows the corresponding binary images obtained by ImageJ software with 16 thresholding methods, respectively. It indicates that most of the thresholding methods work well when the fluorescent spots are bright with a dark background, for example, fluorescent spots #1 and #3. However, when the fluorescent spots are very opaque compared to the background (#2 and #6), or when the background is strong or not uniform (#5), the binary images of many thresholding methods become faint. After comprehensive comparison of these thresholding methods, it is easy to find that the results of the Otsu method, which are marked in red, exhibit much clearer binary images than the others. The Otsu method is a widely used automatic image thresholding method suitable for images with only two classes, foreground, and background [34]. In our case, the image also has only two classes with the fluorescent spots as the foreground and the other area as the background. Thus, the Otsu method was chosen for the smartphone application development.

**Figure 4.**
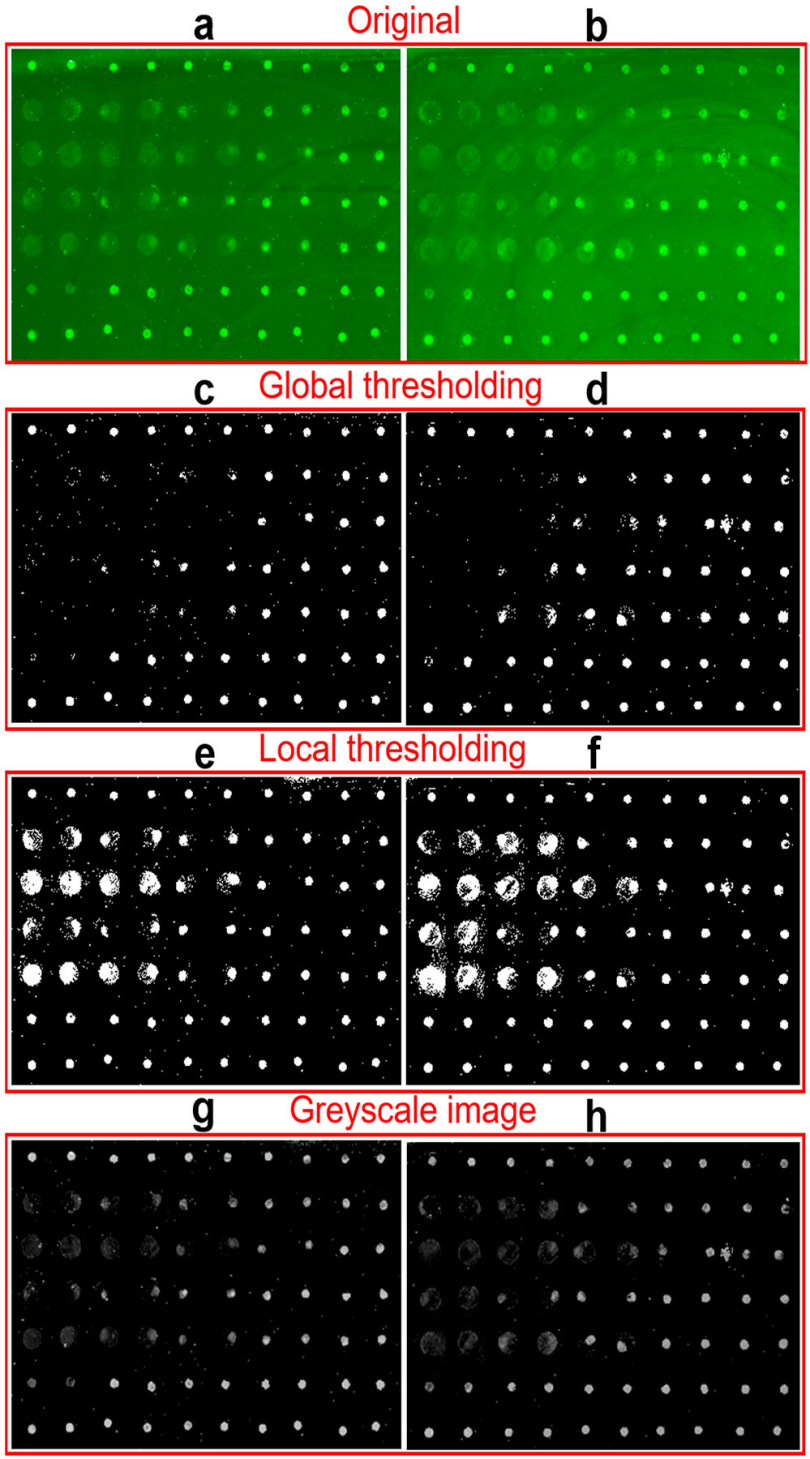
Comparison of global thresholding and local thresholding. (**a**,**b**) The original fluorescent images of two experimental spots matrix. (**c**,**d**) The corresponding binary images of images (**a**,**b**) after global thresholding processing. (**e**,**f**) The binary images of images (**a**,**b**) after local thresholding processing. (**g**,**h**) The corresponding greyscale images of (**a**,**b**) after subtracting local background.

In practical image processing, the sample always includes tens to hundreds of spots with different qualities. What’s more, the images always have background noise which makes the binarization difficult. Thresholding schemes including global and local thresholding are the most popular methods for image segmentation from the background [32,35,36]. Figure 4a,b show two typical original images with strong or non-uniform backgrounds. Here, only 70 points are included in the detection example to demonstrate feasibility of analyzing the protein microarray signals using our newly-developed smartphone application, but hundreds or even more points can be printed and processed as shown in our previous work [11]. Figure 4c-f shows the corresponding binary images processed by global and local thresholding methods to extract the spots from background and locate their positions. The bright pixel in binary images will be included in the calculation of fluorescent intensity. It can be found that many fluorescent spots with both light background and low brightness in Figure 4c,d are missed out by the global thresholding processing after subtracting the strong global threshold [32,36]. On the contrary, the local thresholding images in Figure 4e,f retained all the spots by dividing the images into multiple areas with only one spot inside and subtracting the background individually. It should be mentioned that brightness of spots in binary images Figure 4c-f does not represent the fluorescence intensity. Based on the localization of spots, the fluorescence intensity is calculated from the greyscale images with subtraction of local background in Figure 4g,h. By combining local thresholding and Otsu method binarization, the processing accuracy of images with complex background can be significantly improved.

**Figure 5.**
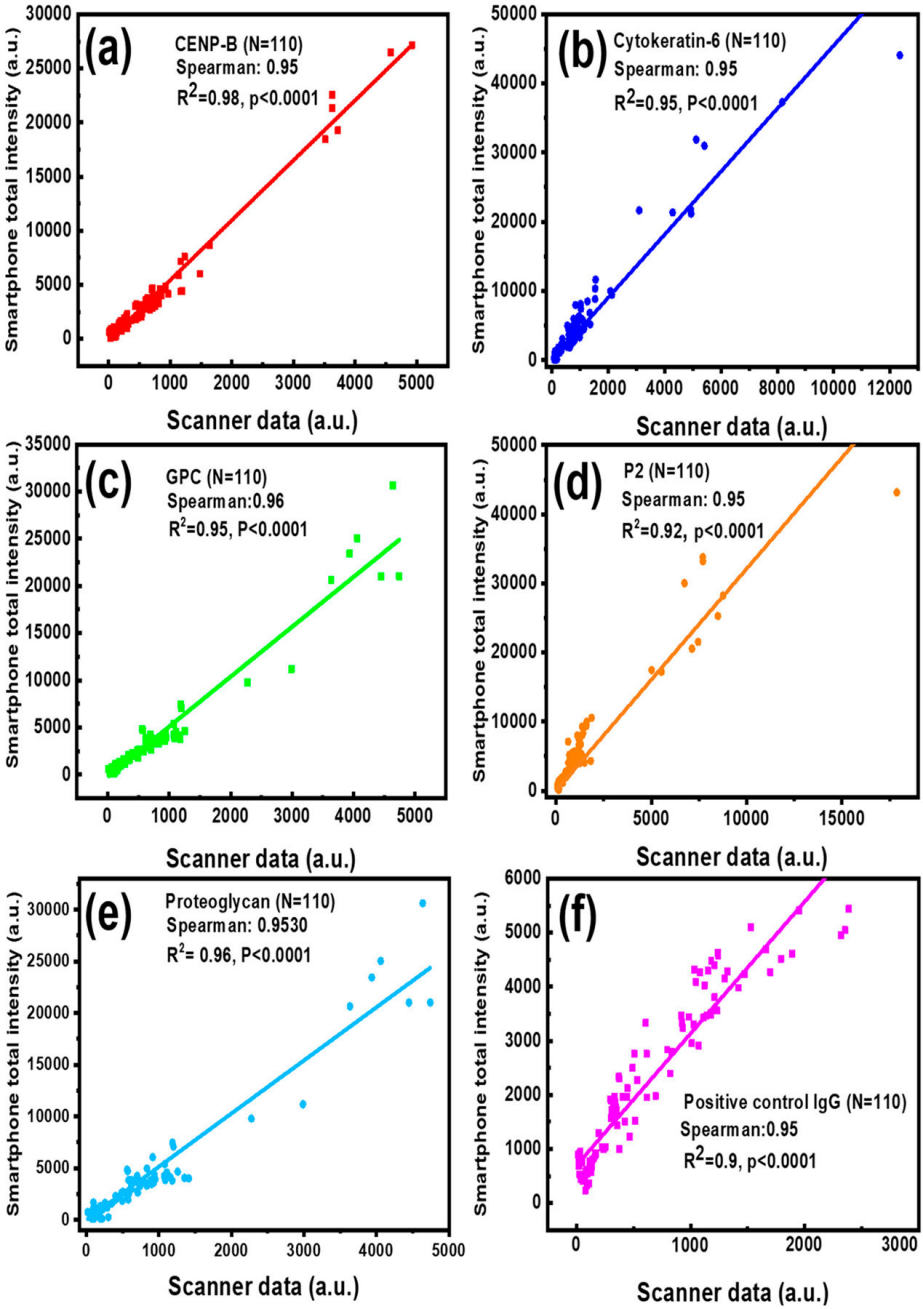
Correlation between the reference data measured by the lab scanner GenePix Pro 7 program and the data obtained by the smartphone application when detecting lupus serum biomarkers: CENP-B (**a**), Cytokeratin-6 autoantibody (**b**), GPC (**c**), P2 autoantibody (**d**), Proteoglycan autoantibody (**e**) and positive control IgG (**f**).

The protein arrays have been widely used for screening autoantibody or autoantigen specificities of different diseases [37-39]. To evaluate the feasibility and reliability of our smartphone-based application in clinic testing, serum samples from lupus nephritis (LN) patients and healthy controls (HC) were tested on an autoantigen microarray where CENP-B, Cytokeratin-6, GPC, P2, and Proteoglycan recombinant proteins were printed, as well as a positive control IgG. This antigen array slide was used for the detection of antigen-specific autoantibody levels. The images from the autoantigen microarray with hundreds of fluorescent spots were processed by a commercial GenePix Pro 7 program installed in the lab scanner and our smartphone application, respectively. For the complete validation of these data, the correlation analysis was performed by calculating the Spearman’s rank correlation coefficient between the two methods. As shown in Figure 5a-f, Spearman’s correlation coefficients between the two methods for the five autoantibodies and positive control IgG were found to be over 0.95 and a linear regression of R^2^ ≥ 0.9, indicating a strong association between our application and the commercial GenePix program. What’s more, the significance of the correlation coefficient *p* values, given by the software, are all smaller than 0.0001, confirming that the correlation of analytical results between the GenePix Pro 7 software and our smartphone app is strong. Here, only several high concentration spots were printed in each sample because they are rarely used in practical detection and higher concentration autoantigen will block the printing needle for the increased viscosity. However, the high overall correlation coefficient (over 0.95) between two methods demonstrates the reliability of the smartphone application in the typical testing concentration range.

The successful detection of autoantibody and autoantigen on array demonstrated that this smartphone software can analyze the signals of various targets but is not limited to a special condition. In the detection spots numbers of microarray, this software is based on the users’ definition of the matrix, making it flexible to deal with hundreds of spots at one time. This flexibility makes it a universal alternative for different conditions, such as the 36 spots matrix of Balsam’s work, 64 samples in the study of Wang et al., and 289 dots in Hedde’s research [10,21,23]. Although the application analyzes microarray images with multiple spots, the correlation between the data of smartphone and lab scanners is very high and comparable with that of specially designed software [8,10,20,21,24]. The above results and performance discussions indicate that our smartphone application is promising in broad microarray image processing [10,20,23-25].

Given the excellent performance of our smartphone-based App in the above autoantigen and antibody microarrays, it is promising that smartphone-based biosensors could significantly improve the selectivity and sensitivity when detecting various disease biomarkers. The antibody is an important component of sensors and has attracted high attention [40]. The antigen-antibody interaction provides excellent sensitivity and specificity and promotes the development of bio-devices that could selectively target and detect various analytes such as biomarkers, cells, and pathogens [41]. However, multiple requirements for the traditional immunosensors and image analysis, such as expensive software and an expert workforce, limit their wide application in the clinical field for point-of-care usage.

As one of the popular optical techniques, fluorometry has attracted significant attention because of several benefits including sensitivity, selectivity, and speed. A cost-efficient fluorescence immunosensor based on a smartphone was fabricated by Yu et al. to quantify amantadine [42]. Moreover, Zeinhom et al. developed a portable fluorescence imaging system using the protein-nanomaterial hybrid technique to detect E. coli in Yoghurt and Egg [43]. The fixed bacteria interacted with the fluorescein-labeled rabbit poly-antibody, which was used as the signal molecule for the detection. Furthermore, quantum dots were also utilized by Ludwig and colleagues to design the fluorescence immunosensor, and the emitted fluorescence light with the target could be detected and analyzed with their custom-designed application [13]. Instead of the sophisticated apparatus and expensive commercial software for the common analytical laboratory device, smartphone-based fluorescent emission signals analysis provides a portable and low-cost platform. It is easier than the conventional method since it does not require any heavy and expensive instruments [44]. Moreover, it provides comparable results with significantly high accuracy when compared to the conventional method, suggesting its high potential to serve as a point-of-care analysis tool in the clinical field.

**Figure 6.**
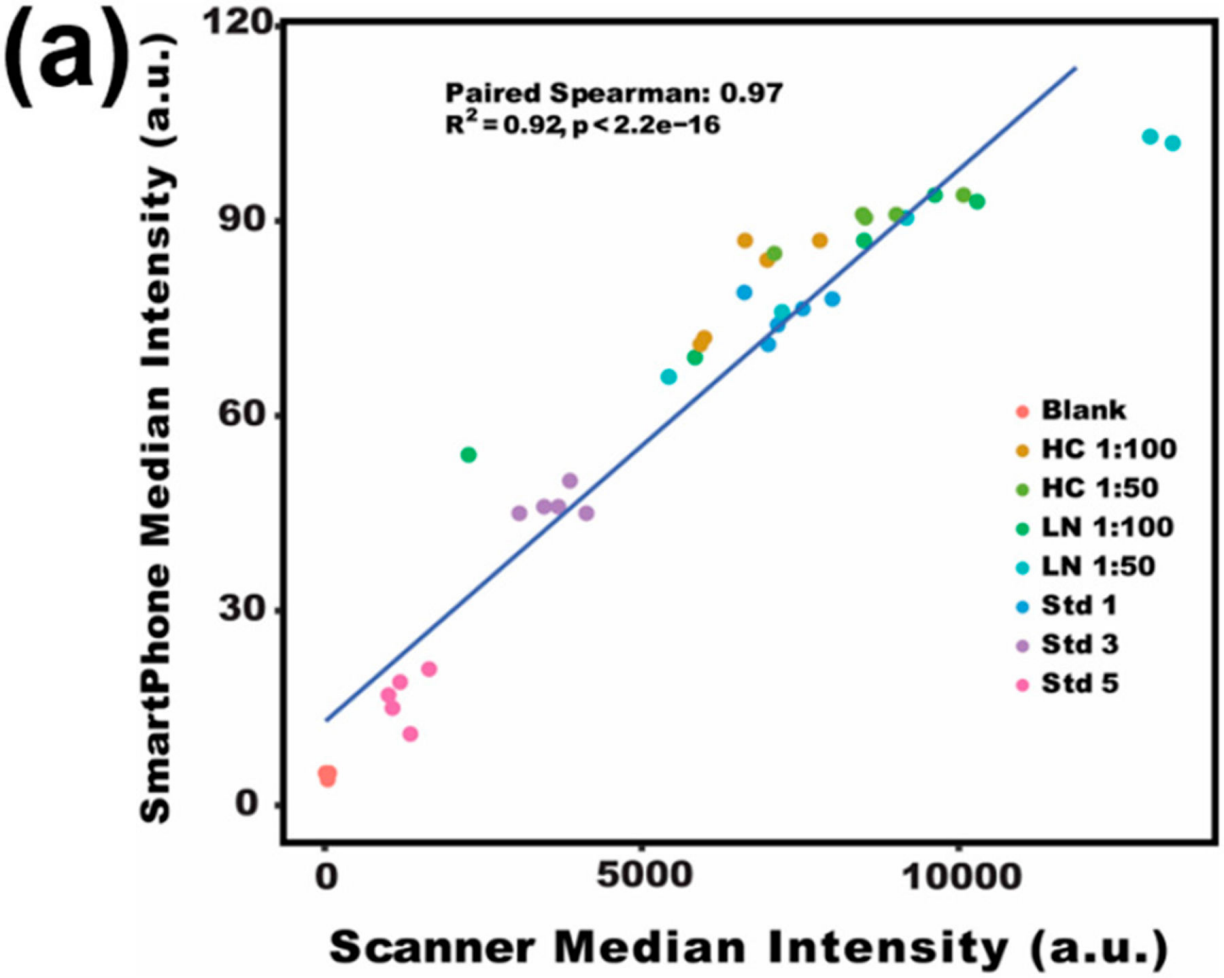

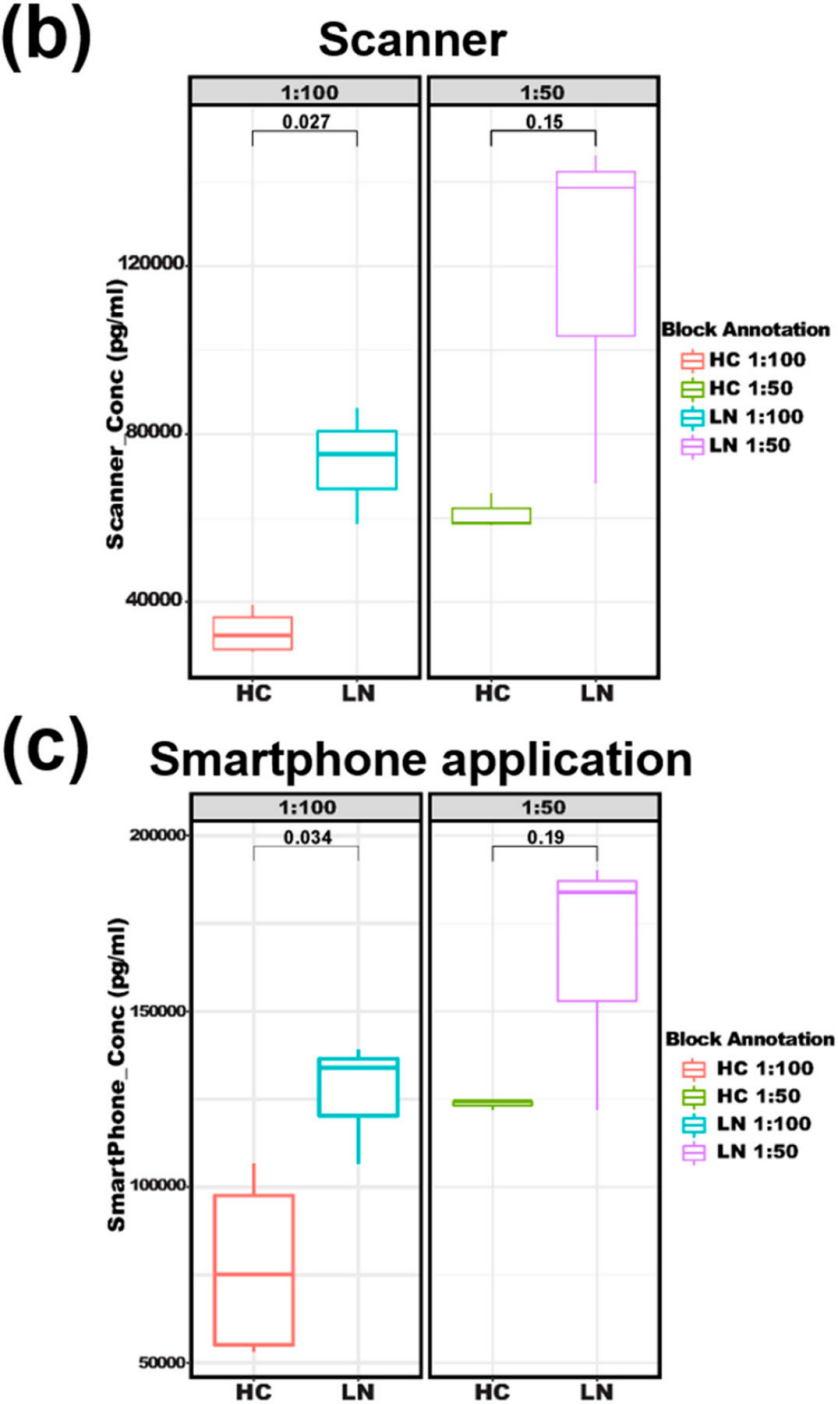
Testing of serum sCD14 concentrations with an antibody microarray. (**a**) Correlation of scanner reads with smartphone reads for sCD14 array results. (**b**) The scanner-based boxplot compares sCD14 levels in LN versus HC at different serum dilutions. (**c**) The smartphone-based boxplot compares sCD14 in LN versus HC at different serum dilutions. HC: healthy control, LN: lupus nephritis, Std: recombinant CD14 protein as a standard, sCD14: soluble CD14.

It is worth noting that by using our smartphone-based app, we were able to accurately detect serum soluble CD14 as a potential disease-activity-related biomarker of lupus [45,46]. sCD14 concentrations in patients with lupus nephritis were reported around 2~10 mg/L in plasma [47,48]. In this assay, CD14 captures the antibody and detection antibody were used to form a sandwich structure with the analyte to detect CD14 in a microarray format. As shown in Figure 6, both strong Spearman correlation (R = 0.97) and high fitness of linear regression (R^2^ = 0.92) were found between the two methods when detecting serum CD14 in lupus patients versus healthy subjects (Figure 6a). For the serum dilution ratio of 1:100 in Figure 6b,c, a significant difference in sCD14 concentration was detected (*p*-value < 0.05) between LN patients and healthy controls by both the smartphone App and GenePix. A slightly larger p-value (0.034 and 0.19 for dilution ratio of 1: 100 and 1: 50, respectively) from the smartphone App than that of GenePix (0.027 and 0.15) is believed to be due to their different data processing algorithms. Overall, lupus is a multifactorial and heterogeneous autoimmune disease with significant pathological consequences. However, the clinical diagnostics of lupus are suboptimal [49]. With the development of omics technologies, more SLE potential biomarkers are being identified, especially from blood, urine, and other body fluids which may provide alternative approaches to associate with lupus manifestations [50]. However, the bottleneck for biomarker-based diagnostics is the difficulty of detecting a panel of biomarkers simultaneously. Fortunately, our protein microarray-based biomarker detection system allows for multiplexing and a high-throughput assay of multiple biomarkers at a time [51]. With the rapid increase of camera resolution to tens of millions and the arbitrary definition of microarray rows and columns in our smartphone application (shown in Figure 2), the throughput can be several hundred and even thousand [8,10,21]. By integrating the array-based detection, portable imager, and user-friendly smartphone application, we may be able to develop the next-generation diagnostic tools for lupus and lupus nephritis.

This work independently designed an open-source smartphone application to reliably analyze autoantigen and antibody microarray data for point-of-care diagnosis, but not a duplication of commercial software on a computer as it is not open and freely accessible. The utilization of free Android platform and universality of this smartphone software reduce the cost prominently. When an imaging accessory is attached, a comprehensive system with scanning, analyzing, and reporting functions will be developed in portable devices. Then optics optimization, operation systems compatibility, and calibration can be conducted to improve the imaging quality, accuracy, and flexibility of the system. Moreover, the data transfer function will be added in the system for communication between smartphones, computers, and servers to share user experience, collect data, and monitor disease activity.

## 4. Conclusions

In this work, we developed a smartphone application that can simultaneously read, analyze, and report the intensities of multiple fluorescent spots in one sample image. The application was compatible with a broad range of devices as it is based on Android Studio open platform, and the graphical user interface makes the operation simple and convenient. The image processing based on a smartphone makes it a universal application for various microarray detecting but is not limited to specific instruments. To verify the efficiency, reliability, and high throughput of this tool, intensities of various antigens with many spots were tested and showed strong positive correlations with that of the commercial scanner. Moreover, the number of array spots can be increased based on requirements since the smartphone application has flexibility to adjust the inputting rows and column indices. More functions, such as data transfer and graph display, can also be further integrated in the future. In addition to the fluorescent microarray detection, this application can also be used in many fields, such as colorimetric sensing [52-54], surface plasmon resonance detection [55], and optical transmission sensing [22], making it a promising and versatile way for POC testing and home diagnosis.

## Data Availability

Data is contained within the article or supplementary materials.
